# From attention-deficit hyperactivity disorder to sporadic Alzheimer’s disease—Wnt/mTOR pathways hypothesis

**DOI:** 10.3389/fnins.2023.1104985

**Published:** 2023-02-16

**Authors:** Edna Grünblatt, Jan Homolak, Ana Babic Perhoc, Virag Davor, Ana Knezovic, Jelena Osmanovic Barilar, Peter Riederer, Susanne Walitza, Christian Tackenberg, Melita Salkovic-Petrisic

**Affiliations:** ^1^Department of Child and Adolescent Psychiatry and Psychotherapy, Psychiatric University Hospital Zurich (PUK), University of Zurich, Zurich, Switzerland; ^2^Neuroscience Center Zurich, University of Zurich and the Swiss Federal Institute of Technology (ETH) Zurich, Zurich, Switzerland; ^3^Zurich Center for Integrative Human Physiology, University of Zurich, Zurich, Switzerland; ^4^Department of Pharmacology and Croatian Institute for Brain Research, University of Zagreb School of Medicine, Zagreb, Croatia; ^5^Department of Psychiatry, Psychosomatics and Psychotherapy, Center of Mental Health, University Hospital Würzburg, Würzburg, Germany; ^6^Department and Research Unit of Psychiatry, Institute of Clinical Research, University of Southern Denmark, Odense, Denmark; ^7^Institute for Regenerative Medicine (IREM), University of Zurich, Schlieren, Switzerland

**Keywords:** Alzheimer’s disease, Wnt/mTOR, cognitive impairment, glucose/insulin, oxidative stress, methylphenidate, attention-deficit hyperactivity disorder (ADHD), mild cognitive impairment (MCI)

## Abstract

Alzheimer’s disease (AD) is the most common neurodegenerative disorder with the majority of patients classified as sporadic AD (sAD), in which etiopathogenesis remains unresolved. Though sAD is argued to be a polygenic disorder, apolipoprotein E (APOE) ε4, was found three decades ago to pose the strongest genetic risk for sAD. Currently, the only clinically approved disease-modifying drugs for AD are aducanumab (Aduhelm) and lecanemab (Leqembi). All other AD treatment options are purely symptomatic with modest benefits. Similarly, attention-deficit hyperactivity disorder (ADHD), is one of the most common neurodevelopmental mental disorders in children and adolescents, acknowledged to persist in adulthood in over 60% of the patients. Moreover, for ADHD whose etiopathogenesis is not completely understood, a large proportion of patients respond well to treatment (first-line psychostimulants, e.g., methylphenidate/MPH), however, no disease-modifying therapy exists. Interestingly, cognitive impairments, executive, and memory deficits seem to be common in ADHD, but also in early stages of mild cognitive impairment (MCI), and dementia, including sAD. Therefore, one of many hypotheses is that ADHD and sAD might have similar origins or that they intercalate with one another, as shown recently that ADHD may be considered a risk factor for sAD. Intriguingly, several overlaps have been shown between the two disorders, e.g., inflammatory activation, oxidative stress, glucose and insulin pathways, wingless-INT/mammalian target of rapamycin (Wnt/mTOR) signaling, and altered lipid metabolism. Indeed, Wnt/mTOR activities were found to be modified by MPH in several ADHD studies. Wnt/mTOR was also found to play a role in sAD and in animal models of the disorder. Moreover, MPH treatment in the MCI phase was shown to be successful for apathy including some improvement in cognition, according to a recent meta-analysis. In several AD animal models, ADHD-like behavioral phenotypes have been observed indicating a possible interconnection between ADHD and AD. In this concept paper, we will discuss the various evidence in human and animal models supporting the hypothesis in which ADHD might increase the risk for sAD, with common involvement of the Wnt/mTOR-pathway leading to lifespan alteration at the neuronal levels.

## 1. Introduction

The orchestration of brain development, maturation, plasticity, repair, and survival is highly complex, in which any imbalance in one or more of these processes may induce disruptions manifested at different stages of life, from the neurodevelopmental disorders at the early stages to neurodegeneration of the brain in late stage (Feltes et al., 2015). These opposite ends of the timeline may appear unrelated, as in the case of attention-deficit hyperactivity disorder (ADHD) and Alzheimer’s disease (AD), which represent mental disorders affecting the early and late ends of lifespan, respectively. However, literature data indicates there are some overlaps between ADHD and AD (Kakuszi et al., 2020; Zhang et al., 2022). In this article, we will lay down the current evidence proposing the involvement of important pathways, participating in many of the above lifelong processes in the brain, i.e., the wingless-INT (Wnt), and the mammalian target of rapamycin (mTOR) (Wnt/mTOR) pathway, as a common link between ADHD and AD, and a target for a drug that can therefore act therapeutically in both diseases (Kovacs et al., 2014; Lee, 2015; Noelanders and Vleminckx, 2017; Palomer et al., 2019; Marchetti et al., 2020; Ishikawa and Ishikawa, 2022).

Before delving deep into our hypothesis, some comparisons between ADHD and AD are presented in Table 1. ADHD is the most common neurodevelopmental mental disorder in children and adolescents that presents as inattentiveness, hyperactivity, impulsivity, and emotional dysregulation (Faraone et al., 2015). Though considered a childhood disorder, in about 60% of the cases it persists into adulthood (Franke et al., 2018; Di Lorenzo et al., 2021; Song et al., 2021). ADHD is characterized by high heritability (77–88%) (Faraone and Larsson, 2019), however, current evidence suggests that the development of the disease is driven by complex interactions of composite polygenic and multiple environmental factors that undermine developmental processes resulting in altered brain connectivity and structure (Castellanos and Tannock, 2002; Faraone et al., 2015). On the other hand, AD is the most common neurodegenerative disorder that poses a substantial socioeconomic burden on society (Sontheimer et al., 2021; Velandia et al., 2022). A fully penetrant autosomal dominant inheritance of amyloid precursor protein (APP), presenilin-1 (PS1), or presenilin-2 (PS2) mutations drives the development of the disease in a small fraction of patients [0.5–1%; familial AD (fAD)], however for the remaining 99% classified as sporadic AD (sAD), the etiopathogenesis remains unresolved (Reitz et al., 2020). Although, sAD seems to be a polygenic disorder, the most prominent locus, apolipoprotein E (APOE), found already three decades ago, was confirmed to have the strongest genetic risk for sAD in the most recent and largest genome-wide association meta-analysis study (meta-GWAS), with APOE ε4 as the risk allele4 (de Rojas et al., 2021; Wightman et al., 2021; Bellenguez et al., 2022). At first glance, no common mechanism seems to link these two diseases together. However, several recent overviews have discussed their complementary phenomena (Feltes et al., 2015; Callahan et al., 2017), and possible genetic overlaps (Garcia-Argibay et al., 2022; Leffa et al., 2022).

**TABLE 1:** Current knowledge in attention-deficit hyperactivity disorder (ADHD) and Alzheimer’s disease (AD)- A comparison.

	ADHD		AD	
Definition	Neurodevelopmental mental disorder	American Psychiatric Association (2013)	–	
	–		Neurodegenerative mental disorder	American Psychiatric Association (2013)
Demographics	Prevalence ca 5% world-wide in child and adolescent	Sayal et al. (2018)	Prevalence ca 0.7% old dementia world-wide in old aged individuals	GBD 2019 Dementia Forecasting Collaborators (2022)
	Persisting into adulthood ca 60% of pediatric ADHD	Fayyad et al. (2017); Franke et al. (2018)	–	
	Childhood: male to female 3:1 Adulthood: male to female 1.6:1	Willcutt (2012); Sayal et al. (2018)	– Old age: male to female 1:1.67	Podcasy and Epperson (2016)
Genetics	n.a.		1% Familial AD (*APP, PS1* and *PS2*)	Serretti et al. (2007)
	Heritability (h^2^) ca 70% and polygenic	Demontis et al. (2019, 2023); Faraone and Larsson (2019)	Heritability (h^2^) ca 58–70% and polygenic including *APOEε4* as risk allele	Sims et al. (2020); de Rojas et al. (2021); Wightman et al. (2021)
Main clinical phenotypes	Inattention, hyperactivity, impulsivity	American Psychiatric Association (2013)	–	
	–		Cognitive decline	American Psychiatric Association (2013)
	Executive dysfunction	Pineda et al. (1998); Willcutt et al. (2005)	Executive dysfunction	Swanberg et al. (2004); Baudic et al. (2006)
Comorbidity	Depression Anxiety ASD Bipolar in adulthood Sleep disorder	Jensen and Steinhausen (2015); Wajszilber et al. (2018); Mayer et al. (2021); Sandstrom et al. (2021); Schiweck et al. (2021)	Depression Anxiety – – Sleep disorder	Zhao et al. (2016); Kuring et al. (2020)
	T2DM Metabolic syndrome Hypertension	Chen et al. (2018); Landau and Pinhas-Hamiel (2019); Wang et al. (2021); Ai et al. (2022)	T2DM Metabolic syndrome Hypertension	Wang et al. (2018); Qin J. et al. (2021); Zuin et al. (2021); Antal et al. (2022)
Treatment	Only reducing symptoms: psychostimulants (e.g., methylphenidate, dexmethylphenidate, amphetamine, dexamphetamine, lisdexamphetamine), non-psychostimulants (e.g., atomoxetine, guanfacine)	Banaschewski et al. (2018)	Only reducing symptoms: acetylcholinesterase (AchE) inhibitors and memantine	National Institute on Aging (2021)
	n.a.		Possible disease-modifying: aducanumab	National Institute on Aging (2021)
Affected brain regions	Cerebral cortex (forebrain) Basal ganglia Amygdala Hippocampus	Hoogman et al. (2017)	Cerebral cortex (medial and superior frontal gyrus) Putamen Amygdala Hippocampus	Li et al. (2012, 2015); Lupton et al. (2016); Roe et al. (2021); Planche et al. (2022)
Current hypothesis to etiopathology	Neuronal maturation delays	Shaw et al. (2007); Hoogman et al. (2017)	–	
	–		Pathological Amyloid-β	Hampel et al. (2021)
	Dopaminergic deficit theory	Gonon (2009)	–	
	–		Pathological Tau forms and neurofibrillary tangles	Hasani et al. (2021)
	–		Cholinergic neuronal damage	Lyness et al. (2003)
	–		APOE cascade hypothesis	Martens et al. (2022)
	Excitatory/inhibitory imbalance theory	Selten et al. (2018)	Excitatory/inhibitory imbalance theory	Shu et al. (2022); van den Berg et al. (2022); Wood et al. (2022)
	Oxidative stress and mitochondria dysfunction	Corona (2020)	Oxidative stress and mitochondria dysfunction	Mittal et al. (2022)
	Neuroinflammation	Misiak et al. (2022)	Neuroinflammation	Kinney et al. (2018)
	Energy metabolism- cerebral glucose hypometabolism	Zametkin et al. (1990)	Energy metabolism- cerebral glucose hypometabolism; brain insulin resistance	Kellar and Craft (2020); Strom et al. (2022)

APOE, apolipoprotein E; ASD, autism spectrum disorder; n.a., not available; –, not relevant; T2DM, Type 2 diabetes mellitus.

In the current paper, we present our hypothesis of the Wnt/mTOR pathway playing a role both in ADHD and sAD pathophysiology, which may explain the recent findings of ADHD as a risk for sAD (Tzeng et al., 2019; Dobrosavljevic et al., 2021; Zhang et al., 2022). Furthermore, we discuss the possible therapeutic potentials of the psychostimulant methylphenidate (MPH) which, by affecting the Wnt/mTOR pathway, might be beneficial in both disorders.

## 2. Current knowledge of Alzheimer’s disease pathology, insulin resistance, and diabetes mellitus

Alzheimer’s disease is a neurodegenerative disorder and the most common form of dementia with an estimated number of 55 million people currently suffering from AD worldwide, predicted to reach 78 million in 2030 and 139 million in 2050 (Alzheimer’s Disease International, 2022). The well-known neuropathological hallmarks of AD are an accumulation of misfolded proteins, amyloid β (Aβ) in the forms of extracellular plaques and hyperphosphorylated Tau protein in the form of neurofibrillary tangles, accompanied by synaptic loss (Sanabria-Castro et al., 2017; Table 1). Less common early onset disease (predominantly fAD) is associated with autosomal dominant missense gene mutations in PS1 and PS2 or APP. On the contrary, the most common, late-onset or sAD form (>95% of all AD cases) is of unknown origin, and is considered polygenic (de Rojas et al., 2020; Wightman et al., 2021; Bellenguez et al., 2022; Table 1). Though the sAD etiopathogenesis is still unclear, several hypotheses in addition to the most prominent, the amyloid hypothesis (Hampel et al., 2021), have been suggested. Recently, sAD has been acknowledged as a metabolic disease with characteristic neurodegenerative processes possibly being caused by brain insulin resistance (BIR) and metabolic dysfunction in the brain (De Felice et al., 2014; Bloom et al., 2018; Kellar and Craft, 2020; Alves et al., 2021). Current epidemiological and environmental studies suggest that type 2 diabetes mellitus (T2DM) increases the risk of developing sAD (Xue et al., 2019; Rebelos et al., 2021). Recently, a polygenic risk score (PRS) for T2DM was found to predict the conversion of amnestic mild cognitive impairment (MCI) to sAD, with shared genes highly expressed in cortical neurons; neuronal development and generation, cell junction and projection, and phosphatidylinositol 3-kinase (PI3K), protein kinase B (Akt) and mitogen-activated protein kinase (MAPK) signaling pathway (Yang et al., 2022). There are many shared pathological findings in the brain of sAD and T2DM patients that should not be overlooked. These include impaired glucose metabolism, impaired insulin signaling, the accumulation of advanced glycation end products, mitochondrial dysfunction, increased inflammation and elevated oxidative stress, which altogether support the hypothesis of sAD as a specific form of metabolic brain disorder (Kubis-Kubiak et al., 2019; Austad et al., 2022; Pakdin et al., 2022).

Brain insulin resistance is characterized by a reduced response to insulin signaling downstream of the insulin receptor (IR) in the brain, consequently leading to metabolic alteration, neurodegeneration, and cognitive impairment (Kullmann et al., 2017; Kellar and Craft, 2020). 18F-fluorodeoxyglucose-positron emission tomography (FDG-PET), revealed that individuals with reduced FDG-PET brain metabolism are prone to a much faster cognitive decline and brain atrophy compared to individuals without significantly impaired FDG-PET uptake (Ou et al., 2019), indicating the importance of glucose hypometabolism and BIR in sAD development. In postmortem studies, sAD patients demonstrate decreased brain insulin levels, diminished levels of IR protein and mRNA, as well as altered levels of components downstream of the IR signaling cascade (Frölich et al., 1998; Bartl et al., 2013a; Riederer et al., 2017). BIR has been analyzed in the hippocampal fields CA1–CA3, the dentate gyrus, and the subiculum, which develop marked AD pathology starting in the early phase of the disease, and in the cerebellar cortex, which develops limited pathology seen only in the late phase of AD (Talbot et al., 2012). The markedly reduced insulin signaling downstream of the IR→IR substrate-1 (IRS-1)→ PI3K signaling pathway was found postmortem in the hippocampal and cerebellar cortex in sAD patients without diabetes (Talbot et al., 2012). The major finding was the elevated Serine phosphorylation of IRS-1 on epitopes pS616 and pS636 in the hippocampal and cerebellar cortex, which is a feature of insulin resistance in peripheral tissues (Talbot et al., 2012; Yarchoan et al., 2014).

The state of BIR, clinically defined as failure of insulin (delivered into the brain *via* the intranasal route to bypass the blood-brain barrier) to elicit a neuroimaging (FDG-PET or functional magnetic resonance imaging) or neurophysiological (e.g., electroencephalography) appears to be an early and common feature in human AD patients (Heni et al., 2015; Kullmann et al., 2016; Kellar and Craft, 2020). BIR can lead to energy misbalance manifesting as mitochondrial dysfunction and an increase in oxidative stress (Reddy, 2014). This may shed new light on the previously proposed hypotheses of sAD, which have emphasized the involvement of oxidative stress and mitochondria dysfunction as well as neuroinflammation in the disease etiopathogenesis (Munch et al., 1998; Bachiller et al., 2018; Monterey et al., 2021). As the mitochondria are the power suppliers of the cell, their damage observed in sAD can be deleterious for neurons, astrocytes, and microglia. The decrease in functionality and changes in the morphology of mitochondria is observed postmortem in the brains of AD patients (Moreira, 2012; Macdonald et al., 2018). Altogether, different studies found a decrease in complex I, III, and IV, as well as the decreased expression of subunits from all complexes in the entorhinal cortex of AD patients postmortem (Kim et al., 2000; Armand-Ugon et al., 2017; Macdonald et al., 2018; Holper et al., 2019). All of the above leads to impaired oxidative phosphorylation, decreased production of ATP, and an increase in oxidative stress, features well documented in AD (Wang et al., 2014; Flannery and Trushina, 2019). Additionally, changes in the mitochondrial turnover (fusion and fission) were detected, causing the irregular distribution and function of mitochondria in neurons and microglia (Wilkins et al., 2017). BIR and consequent mitochondrial dysfunction lead to energy deprivation and oxidative stress, causing neuronal damage and activation of astrocytes and microglial cells (Wilkins et al., 2017). Dysregulated microglial and astrocyte function and alterations in their morphology have been related to inflammatory changes observed in sAD (Damani et al., 2011; Mosher and Wyss-Coray, 2014; Monterey et al., 2021). Moreover, aged astrocytes and microglia show altered responses to extracellular ATP signals compared to young cells (Damani et al., 2011; Monterey et al., 2021). Degenerated neurons appear to be surrounded by activated astrocytes and microglia in aging and neurodegenerative diseases (Bachiller et al., 2018; Monterey et al., 2021). Eventually, damaged astrocytes and microglia also undergo metabolic reprogramming due to glucose deprivation, shifting their glucose preference to fatty acids for energy production. This profound metabolic change can be directly linked to oxidative stress and inflammation (Flowers et al., 2017; Monterey et al., 2021).

So far, the anti-Aβ antibodies aducanumab and lecanemab are the only clinically approved disease-modifying drugs for the treatment of AD. Both were recently granted accelerated approval by the U.S. Food and Drug Administration (U.S. Food & Drugs Administration, 2021, 2023). This procedure allows for earlier approval of drugs to treat serious conditions and highlights the unmet medical need for AD treatment options. Thus, there is still the demand for further effective therapies for sAD and involvement of BIR, together with the integration of all of these changes found in sAD, might be crucial in developing novel prevention and treatment strategies.

## 3. Attention-deficit hyperactivity disorder current genetic and etiology hypothesis

As indicated in the introduction, ADHD is a highly heritable neurodevelopmental disorder (Faraone and Larsson, 2019), currently known to be associated with a polygenetic predisposition. In the most recent GWAS, including a total of 38′691 ADHD patients and 186′843 controls, 27 genome-wide significant loci were found to associate with ADHD risk (Demontis et al., 2023). As found previously using neuroimaging meta-analysis (Hoogman et al., 2017), frontal cortex and midbrain dopaminergic neurons were highly associated with ADHD genes (Demontis et al., 2023). Indeed, ADHD was found to be highly polygenic, with around seven thousand gene variants explaining 90% of the single nucleotide polymorphism (SNP) heritability (h^2^) (Demontis et al., 2023). Moreover, gene enrichment analysis found enrichment in genes upregulated during early embryonic brain development as well as genes of cognition-related phenotypes (Demontis et al., 2023). As found in the previous GWAS results (Demontis et al., 2019), as well as in the current large meta-GWAS, several genes (e.g., *DUSP6, SEMA6D, ST3GAL3, FOXP1* and *FOXP2*, and *SORCS3*) linked to the Wnt pathways (canonical and non-canonical) were found to be associated with ADHD (Demontis et al., 2023). Interestingly, some of these genes were also found to associate with dementia or/and with pathological hallmarks of sAD (Liao et al., 2018; Balabanski et al., 2021; Blue et al., 2021; Tang et al., 2021).

Although the etiopathology of the disorder is still unknown, several hypotheses have been suggested (Table 1), proposing factors leading to neurodevelopmental delays observed in ADHD patients (Shaw et al., 2007; Hoogman et al., 2017). As psychostimulants (first-line ADHD treatment; e.g., MPH, amphetamine) (Banaschewski et al., 2018), demonstrate large effect sizes, the dopaminergic deficit theory has been studied for many years (Gonon, 2009). However, the paradoxical calming effects of psychostimulants in ADHD have still not been fully understood (Robbins and Sahakian, 1979; Harris et al., 2022). Recently, the excitatory/inhibitory imbalance theory has gained interest (Selten et al., 2018), in which GABAergic and parvalbumin-interneurons are hypothesized to play a role in some of the circuits (Bakhtiari et al., 2012; Morello et al., 2020; Sousa et al., 2022). Two hypotheses, extensively studied, are the involvement of oxidative stress (Corona, 2020), particularly mitochondrial dysfunction, and the neuroinflammatory hypothesis (Corona, 2020; Misiak et al., 2022). Both may be a result of long-lasting alterations in neurodevelopmental processes, but may also be one of the etiopathogenic factors. Partially linked with mitochondrial dysfunction, the energy metabolism imbalance in ADHD has been discussed as a possible source of neurodevelopmental delays (Cannon Homaei et al., 2022; Foschiera et al., 2022; Radtke et al., 2022). Cerebral glucose hypometabolism has been found in childhood-onset adult ADHD patients (Zametkin et al., 1990), however, this may also be a consequence of deficits in impulse control leading to metabolic syndrome (di Girolamo et al., 2022). Indeed, a recent meta-analysis found a bidirectional association between ADHD and T2DM (Ai et al., 2022), pointing to long-lasting BIR effects. Interestingly, some significant genetic correlations with insulin-related phenotypes were found for ADHD and AD that provide the foundations for the hypothesis of insulinopathies in the brains of such disorders (Fanelli et al., 2022). Lastly, the involvement of Wnt/mTOR pathways in ADHD has been hypothesized (Yde Ohki et al., 2020), with evidence at the genetic, molecular, and pharmacological levels. This hypothesis will be discussed in more detail in the Wnt/mTOR chapter.

In summary, although an effective treatment exists for ADHD, the long-term effects of drug treatment, the consequences of persistent ADHD in adult patients, as well as the etiopathology of the disorder are not fully understood. Nevertheless, metabolic and Wnt/mTOR pathway alterations may be a common factor in ADHD and AD, and a potential target for preventive measures in both diseases.

## 4. Epidemiological evidence for attention-deficit hyperactivity disorder risk for dementia

Epidemiological studies on the link between ADHD and dementia face several prominent methodological challenges – e.g., the required duration of the follow-up in prospective cohorts from childhood to very old age and substantial spatiotemporal variability in diagnostic criteria and therapeutic guidelines (particularly for ADHD). Nevertheless, although there are only a handful of studies investigating the association between ADHD and AD in humans, some evidence point to a possible link suggesting that ADHD might increase the risk for AD. Earlier clinical research on the frequency of ADHD in the aging population suggested that attentional deficits in geriatric subjects with cognitive impairment might not necessarily imply the existence of an underlying neurodegenerative disorder (Ivanchak et al., 2011). However, recent epidemiological and genetic studies indicate that ADHD is a potential risk factor for sAD and MCI (Fluegge and Fluegge, 2018; Tzeng et al., 2019; Dobrosavljevic et al., 2021). A recent multi-generation nationwide cohort study from Sweden provided strong evidence that ADHD is associated with AD (and other dementias) across generations (Zhang et al., 2022), indicating that elucidation of the overlapping molecular pathways in models of ADHD and AD may uncover relevant etiopathogenic mechanisms and reveal novel drug targets. Literature data on factors that might reduce this risk indicates that adjustment for psychiatric disorders (depression, anxiety, substance use disorder, and bipolar disorder) substantially attenuated the associations (Dobrosavljevic et al., 2021). Conversely, there is inconsistency regarding metabolic disorders like T2DM, obesity, and hypertension reported to have both a limited impact (Dobrosavljevic et al., 2021) and a strong risk-increasing effect (Fluegge and Fluegge, 2018). A recent study (Kakuszi et al., 2020), provided findings consistent with the “last in, first out” hypothesis, which refers to a mirroring pattern of brain development and aging, i.e., relatively late-developing brain regions with age become the early degenerating ones, indicating that, in addition to delayed neurodevelopment, ADHD may also be associated with a premature age-related deterioration. On the other hand, in a very small longitudinal study of 6 ADHD adults with an average of 135 months of follow-up data, no memory decline was observed, however, this might be due to the small sample size and the effect of medication (3/6 participants were taking MPH and 5/6 were taking unspecified antidepressants) not taken into account (Callahan et al., 2021). The same group conducted cognitive and neuroimaging assessments of adults (age 50–85 years) with ADHD (*n* = 40), *MCI* = (*n* = 29), and controls (*N* = 37) (Callahan et al., 2022). In the study, the authors reported memory impairment in both ADHD and MCI groups, however, the first was due to an encoding deficit (frontal lobe thinning) while the second was due to a storage deficit (smaller hippocampi). The authors concluded that the resembling phenotype was caused by distinct pathophysiological factors (Callahan et al., 2022). However, some limitations should be taken into account—namely the significant age differences between ADHD and MCI (mean age was 64 and 73.7, respectively), as well as sex differences with a greater proportion of females in the MCI group (72.4% in comparison with 52.5% in the ADHD group), which might play a role in the current findings. Mendonca et al. (2021) reported in a cross-sectional study of older patients with ADHD (*n* = 26), MCI (*n* = 40) and controls (*n* = 41), that ADHD individuals have poorer performance than controls in episodic memory and executive function. These findings were comparable between MCI and ADHD for all domains, which, for clinicians, may result in misdiagnosis (Mendonca et al., 2021). Then again, the study by Leffa et al. (2022) in which 212 cognitively healthy controls were followed up for 6 years, including baseline and longitudinal AD biomarkers assessment (e.g., amyloid-β PET, MRI, cognitive assessments etc.), showed an association between higher ADHD-PRS and cognitive decline. Interestingly, a combined effect of brain Aβ deposits and a high ADHD-PRS [that predicted longitudinal increases in cerebrospinal fluid (CSF) phosphorylated-Tau_131_] score demonstrated a larger effect on cognitive dysfunction than each did individually.

The current epidemiological and clinical association studies on ADHD, MCI, and AD, provide some evidence that those suffering from ADHD may be at an increased risk for the development of MCI and, thereafter, AD, possibly mediated by a common mechanism. However, there is still a great need for studies that may elucidate common pathways and provide an explanation for the temporal pattern of occurrence of ADHD, MCI, and AD in long-lasting longitudinal prospective cohorts.

## 5. Wnt/mTOR pathways evidence in Alzheimer’s disease and attention-deficit hyperactivity disorder

**FIGURE 1:**
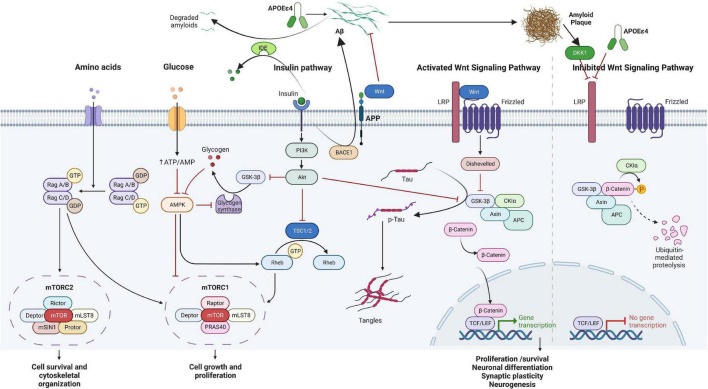
Overview of the Wnt/β-catenin/mTOR signaling pathway hypothesized to be involved in attention-deficit hyperactivity disorder (ADHD) and Alzheimer’s disease (AD) over the lifespan. The two pathways work in a concomitant manner with some common molecules, including energy metabolism and insulin signaling. In the short term, disruption in the pathways may influence growth, differentiation, and synaptic plasticity, while in the long-term, accumulation of p-Tau and amyloid plaques may lead to cell death and decreased neurogenesis. Aβ, amyloid β; Akt, protein kinase B; AMP, adenosine monophosphate; AMPK, adenosine monophosphate-activated protein kinase; APC, adenomatous polyposis coli; APOE, apolipoprotein; APP, amyloid precursor protein; ATP, adenosine triphosphate; DKK1, Dickkopf WNT signaling pathway inhibitor 1; GDP, guanosine diphosphate; GSK3β, glycogen synthase kinase-3β; GTP, guanosine-5′-triphosphate; IDE, insulin-degrading enzyme; LRP, LDL receptor related protein; MAPK, mitogen-activated protein kinase; mTOR, mammalian target of rapamycin; PI3K, phosphatidylinositol 3-kinase; Tau, tubulin associated unit; TCF/LEF, T cell factor/lymphoid enhancer factor family; TSC1/2, tuberous sclerosis proteins 1 and 2; Wnt, wingless-INT. The figure was created using BioRender (https://biorender.com/).

Recent evidence suggests the dysregulation of the Wnt/mTOR signaling pathways as a potential common mechanism in the etiopathogenesis of both ADHD and AD (Boonen et al., 2009; Inestrosa and Varela-Nallar, 2014; Tramutola et al., 2015; Tapia-Rojas and Inestrosa, 2018; Yde Ohki et al., 2020; Perluigi et al., 2021; Nagu et al., 2022; Narvaes and Furini, 2022). Based on the involvement of β-catenin, Wnt signaling can be generally divided into the canonical (β-catenin-dependent) and non-canonical (β-catenin-independent) pathway, activated by binding of different Wnt proteins to their receptors (Wan et al., 2014), which all play an important role in modulation of physiological processes crucial for both developing and mature brain (Jia et al., 2019). During development, Wnt signaling regulates the balance between the proliferation and differentiation of neuronal progenitor and precursor cells (Noelanders and Vleminckx, 2017). In the mature brain, it affects neuronal stem cell proliferation and differentiation (Bengoa-Vergniory and Kypta, 2015). The Wnt pathway has a positive developmental role in the maturation of dendrites and dendritic spines (Hussaini et al., 2014) and an additional role in neurotransmission. Interestingly, recent results also demonstrate that at least some of the effects of Wnt on axonal and dendritic growth may be mediated by APP (Liu et al., 2021). The mTOR pathway plays a key role in maintaining energy homeostasis by regulating nutrient availability and cellular stress information, both intracellularly and extracellularly (Mannaa et al., 2013). Both signaling pathways have some common effectors, like glycogen synthase kinase-3β (GSK3β), which independently participates in both signaling cascades regulating different cellular processes (Kaidanovich-Beilin and Woodgett, 2011). In addition, Wnt ligands can regulate mTOR and insulin signaling pathways (Ackers and Malgor, 2018). The involvement of the aforementioned pathways in ADHD and AD is discussed in the following text (see overview Figure 1).

### 5.1. Canonical Wnt pathway

A pivotal role of Wnt signaling in physiological processes implies that its imbalance may be significant in the pathophysiology of both neurodevelopmental disorders including ADHD (Yde Ohki et al., 2020) and neurodegenerative disorders such as AD (Boonen et al., 2009; Inestrosa and Varela-Nallar, 2014). Therefore, the downstream components of Wnt signaling might be important candidates in both disorders. In the Wnt canonical signaling, activation of the pathway occurs by the binding of Wnt glycoproteins to Frizzled receptors and the lipoprotein receptor-related protein 5 (LRP5) or LRP6 co-receptor, which determine the downstream signaling cascade by the intracellular level and phosphorylation status of β-catenin, modulated by GSK3β (Liu et al., 2002; Jia et al., 2019). In the presence of Dickkopf-1 (Dkk1), a Wnt-inhibitor, GSK3β phosphorylates β-catenin and thus targets it for rapid ubiquitin-dependent degradation by a “destruction complex,” consequently resulting in a low cellular level of β-catenin (Wu and Pan, 2010). On the other hand, activation of Wnt signaling leads to inhibition of GSK3β, disassembly of the β-catenin-containing “destruction complex,” and release of β-catenin, which consequently accumulates and stabilizes in the cytosol, and is then translocated into the nucleus triggering expression of target genes crucial for neuronal survival, neurogenesis, and synaptic plasticity (Jia et al., 2019).

Increased activity of GSK3β has been found in the brain of sAD patients (Llorens-Martin et al., 2014) and increased activation of GSK3β is inversely associated with a significant decrease in β-catenin levels in the prefrontal cortex of AD patients (Folke et al., 2019). Decreased β-catenin levels are unable to suppress the transcription of the β-site APP cleaving enzyme (BACE1), consequently promoting Aβ production and aggregation (Parr et al., 2015), while accumulation of Aβ stimulates the activation of the endogenous Wnt pathway inhibitor, Dkk1, contributing to the decreased β-catenin-dependent triggering of various genes’ expression (Caricasole et al., 2004). Furthermore, since GSK3β is the main kinase involved in the phosphorylation of the Tau protein (Hernandez et al., 2013), a convincing body of evidence indicates that hyperactivity of GSK3β is linked to pathological Tau hyperphosphorylation (Llorens-Martin et al., 2014), thus supporting a link between dysfunctional Wnt/β-catenin signaling with the two major AD hallmarks (Jia et al., 2019). Several AD susceptibility genes are linked to aberrant Wnt signaling, such as APOE ε4, a major genetic risk factor for late-onset sAD, which inhibits canonical Wnt signaling in cell lines (Caruso et al., 2006). Liu et al. (2021) recently reported a direct interaction between Wnt (Wnt3a and Wnt5a) with the cysteine-rich domain in the extracellular portion of APP, a genetic risk factor for fAD. Binding of Wnt3a promoted APP stability, while Wnt5a reduced APP by stimulating lysosomal degradation (Liu et al., 2021). It was shown that both Frizzled1 and Frizzled7 are downregulated in early human AD stages, as well as in the hAPP^NLGF/NLGF^ mouse model (depicting a knock-in of the hAPP Swedish mutation of a fAD), and concomitantly increase Sirtuin2-induced deacetylation (Palomer et al., 2022). Inhibiting Sirt2 *in vivo* and *in vitro* rescued Frizzled expression and synaptic loss (Palomer et al., 2022). The Wnt pathway plays an important role in the regulation of brain insulin signaling. Activation of Wnt upregulates brain-derived insulin in the hypothalamus (Lee et al., 2016) and restores insulin sensitivity in insulin-resistant neurons (Tian et al., 2021). In the rat model of sAD induced by intracerebroventricular streptozotocin (STZ-icv), dysregulation of the IR-PI3K-Akt signaling pathway is associated with increased activity of GSK3β (Barilar et al., 2015). In the STZ-icv mice, increased activity of GSK3β in the hippocampus was accompanied with increased β-catenin (Qi et al., 2021) and decreased Wnt3a and β-catenin (Salem et al., 2021). Furthermore, a recent study modeling sAD using induced pluripotent stem cell (iPSC)-derived cortical neurons from sAD patients and controls, and comparing them to postmortem brain samples from sAD and controls, found at the transcriptomic level, after mapping the findings to the Kyoto Encyclopedia of Genes and Genomes (KEGG) AD map, that pathways of the PI3K and the Wnt-mediated activation of Disheveled, a key component of Wnt signaling, are altered (Verheijen et al., 2022). This provides further support for the involvement of the IR and Wnt pathways in sAD even at an early stage of sAD as iPSC-derived neurons usually represent less mature aging neurons.

Dysregulation in the canonical Wnt signaling pathway in ADHD and its alteration after MPH therapy was confirmed by various researchers as reviewed elsewhere (Yde Ohki et al., 2020). In a mouse model overexpressing the thyroid hormone-responsive protein with defining characteristics of ADHD, a proteomic analysis of the hippocampus found an altered network of proteins involved in Wnt signaling; catenin β1 was found to be upregulated, and hippocampal gene expression of Wnt ligands, inhibitors, receptors, and co-receptors: i.e., significant reductions of the *Wnt7a* gene, as well as upregulation of Wnt inhibitors *Dkk4* and *Igfbp5* and enhanced *Lrp6* expression was found (Custodio et al., 2023). In humans, a significant association was found between sex-specific genetic variations of *LRP5* or *LRP6* co-receptors and child and adolescent ADHD (Grünblatt et al., 2019). As already mentioned in the previous chapter, in a large meta-GWAS, several genes (e.g., *DUSP6, SEMA6D, ST3GAL3, FOXP1* and *FOXP2*, and *SORCS3*) linked to the Wnt-pathways (canonical and non-canonical) were found to be associated with ADHD, further supporting a role of this pathway (Demontis et al., 2023). In a study of Chinese families with a child diagnosed with ADHD, analysis of whole-genome sequencing data showed an increased frequency of single nucleotide variants in several ADHD-susceptible genes, including *LRP6* (Li et al., 2022). Recent comprehensive analysis of mononuclear blood transcriptomic data of 270 ADHD and 279 controls, revealed enrichment of genes involved in the β-catenin-T-cell factors (TCF) complex assembly, AD as well as insulin signaling pathways (Cabana-Dominguez et al., 2022). Interestingly, the *FZD1* gene was significantly down-regulated in ADHD patients compared to controls, similarly to the reported reduction in sAD [internal communication with Cabana-Dominguez et al. (2022)]. Finally, it was also found that MPH enhances neuronal differentiation and reduces proliferation in human SH-SY5Y-cells through activation of the Wnt/β-catenin pathway (Grünblatt et al., 2018). On the other hand, differential regulation by prolonged MPH treatment was found in GSK3β signaling responses in different brain regions (Mines and Jope, 2012; Mines et al., 2013).

### 5.2. mTOR pathway

The serine/threonine kinase mTOR is present in two structurally and functionally distinct protein complexes referred to as mTOR complex 1 (mTORC1) and mTOR complex 2 (mTORC2). mTORC1 integrates signals from multiple growth factors, nutrients, and energy supply, to promote cell growth when energy is sufficient, and catabolism in the absence of nutrients. The mTOR pathway has been found to hold a central role in a variety of cell processes, ranging from promoting protein synthesis to determining the extent of autophagy, and is consequently largely implicated in disease; dysregulation of the pathway has been confirmed in the aging process, cancer, and metabolic disorders like diabetes (Saxton and Sabatini, 2017). mTORC1 is composed of mTOR, the scaffolding protein raptor (regulatory associated protein of TOR), the GTPase β-subunit-like protein (GβL/mLST8) and deptor (Dowling et al., 2010). mTORC2 mainly controls cell proliferation and survival and is comprised of mTOR, Rictor, GβL, Proline Rich 5 (PRR5/Protor), deptor, and mammalian stress-activated protein kinase interacting protein (SIN1) (Zou et al., 2020). mTOR is involved in many signaling pathways in the body and coordinates or interacts with several upstream signal components, including insulin, growth factors, 5-AMP-activated protein kinase (AMPK), PI3K/Akt, and GSK3 (Cai et al., 2015). Due to its extensive presence in different cellular processes, changes in the function of the mTOR pathway activity significantly alter cell homeostasis. On the one hand, decreases in protein synthesis rates driven by inhibition of the mTOR pathway governing mRNA translation may allow for improved cellular proteostasis, whereas increases in the activity of the autophagy-lysosomal pathway due to mTOR inhibition leads to degradation of damaged organelles and macromolecules (Johnson et al., 2015). Indeed, diminished activation of the PI3K/Akt/mTOR pathway was found to augment longevity in mice. Likewise, increased insulin sensitivity in centenarians has been associated with decreased mTOR activity (Sharp and Bartke, 2005). Therefore, longevity appears to be associated with the reduced activity of insulin or insulin-like growth factor (IGF)-mediated PI3K/Akt/mTOR pathway, suggesting that these signaling cascades may be important targets for pharmacological manipulation (Blagosklonny, 2006). However, since insulin and IGF-1 activate mTOR through the PI3K pathway, ultimately regulating cell growth and proliferation in neuronal progenitor cells and neuronal differentiation (Han et al., 2008), as well as synaptic plasticity, glucose, and lipid metabolism, and protein homeostasis (Bedse et al., 2015), chronic inhibition of the pathway is bound to result in deleterious effects, including carcinogenesis and metabolic dysfunction (Ali et al., 2022). Therefore, finely balanced activation of mTOR is of equal interest, e.g., in the context of neurodegeneration, intact mTOR signaling is vital for long-lasting forms of synaptic plasticity and hippocampal memory consolidation and maintenance, by supporting protein synthesis in dendrites and their synapses (Querfurth and Lee, 2021).

Abnormal regulation of the mTOR-pathway is seen in the brain of individuals affected by AD as well as various tissues of patients with T2DM (Mannaa et al., 2013). Although many antidiabetic drugs can affect the mTOR pathway (and introduce bias), current evidence supports the hypothesis that its hyperactivation plays the role in the etiopathogenesis of the disease (Jia et al., 2014; Ong et al., 2016; Ali et al., 2017; Guillen and Benito, 2018). Furthermore, increased levels of pIRS1^Ser636^ and pGSK3β^Ser9^, and hyperactivation of the Akt/mTOR/p70S6K pathway was reported in neuronal-derived extracellular vesicles from patients with Down syndrome (Perluigi et al., 2022) with an increased risk of AD (Zigman and Lott, 2007). Hyperactivation of the mTOR pathway in the brain of AD patients was shown in a number of studies (Uddin et al., 2020), however on the contrary, reduced mTOR signaling was also reported in patients as well as animal models and cell cultures (Lafay-Chebassier et al., 2005). In sAD, neuronal resistance to insulin and IGF-1 is promoted by the over-activation of the PI3K/mTOR axis *via* a negative feedback mechanism that inhibits IRS1 (Tramutola et al., 2015). Moreover, neuronal insulin resistance is also associated with neuroinflammation *via* the tumor necrosis factor-α (TNFα) /c-Jun N-terminal kinases (JNK) pathway, which, in AD, is activated by Aβ oligomers and misfolded Tau that consequently alter the IRS1/mTOR signaling pathway (Liang et al., 2019).

Animal models provide further evidence of the ambiguous role of mTOR activation/inhibition in neurodegeneration and AD. Brain gene expression of several components of the mTOR complex was found to be downregulated in both transgenic (3xTg-AD) and non-transgenic (STZ-icv rat and mice) AD models (Chen et al., 2012; Qin G. et al., 2021). Downregulation of mTOR signaling was found to mediate impairment in synaptic plasticity of the Tg2576 mouse model and increasing mTOR signaling was found to be protective against Aβ-related impairment in long-term potentiation (Ma et al., 2010). In pre-symptomatic and middle-aged APPSwe/PS1ΔE9 (APP/PS1) mice, generation of reactive oxygen species was found to lead to oxidative modification of Akt1 in the synapse resulting in reduction of Akt1-mTOR signaling and deficiency in activity-dependent protein translation; moreover, a similar attenuation of synaptoneurosomal protein translation was found in postmortem AD brains (Ahmad et al., 2017). In cell cultures, STZ-injured oligodendrocytes showed significantly lower expression of PI3K, Akt, p-Akt, mTOR, and p-mTOR proteins than the control group (Liu et al., 2020).

Conversely, inhibition of mTOR was studied for the mitigation of AD symptoms in different AD models. Improvement of cognitive functions and reduction of cortical Aβ levels were found in hAPP(J20) mice after rapamycin (canonical mTOR inhibitor) treatment, along with restoration of neurovascular coupling (Van Skike et al., 2021). Increased hippocampal expression of p-mTOR was reported in the STZ-icv model of AD (El Sayed et al., 2021), while mTOR inhibition by everolimus showed improvements in cognition, as well as lowered TNFα levels in the brain accompanied by increases of insulin and IGF-1 levels and correction of STZ-icv-induced alterations of mRNA expression of PI3K/Akt/mTOR pathway genes (Bansal et al., 2021).

The mTOR pathway’s ubiquitous presence in cell processes implies a role in other disorders, including ADHD. Large-scale computational analysis of the S-nitrosylation proteome pointed to the mTORC1 signaling pathway as one of the shared molecular mechanisms between the autism spectrum disorder (ASD) animal model (InsG3680) and P301S AD mouse model (Mencer et al., 2021). As ADHD is the most common comorbidity in ASD patients (Hours et al., 2022), and ADHD is associated with sAD and any dementia across generations (Zhang et al., 2022), the mTOR pathway may be one of special interest in linking these two disorders. A meta-analysis of GWAS data by Rovira et al. (2020) identified nine new loci indicating a common genetic basis for ADHD in childhood and persistent ADHD in adults, among which was also the *FRAT1/FRAT2* gene regulating the Wnt signaling pathway. In a multi-step analysis aimed to identify and characterize modules of co-expressed genes associated with ADHD using data from peripheral blood mononuclear cells, both *RICTOR* and *MTOR* genes were found to be significantly upregulated in ADHD patients compared to controls further internal discussion with authors regarding data presented in Cabana-Dominguez et al. (2022). In addition, it has been shown by Schmitz et al. (2019) that the Akt-mTOR pathway was affected by MPH treatment in PC12 cells. Short-term MPH treatment decreased pAkt(Thr308)/Akt, p-mTOR/mTOR, and pS6K/S6K ratios, as well as pFoxO1 levels, although long-term treatment increased pAkt(Thr308)/Akt, pmTOR/mTOR and pGSK-3β/GSK-3β ratios (Schmitz et al., 2019). A study of ADHD-susceptible variants by Li et al. (2022) identified genes corresponding to single-nucleotide variants labeled as possibly or probably damaging including *CACNA1H, PKD1, DYNC2H1, LRP6*, and *RGS11*. The *CACNA1H* gene encodes for the α1-subunit of the T-type low voltage-dependent calcium (Ca2+) channel Cav3.2 (Leresche and Lambert, 2017), and mTORC1 pathway regulation may be driven by intracellular Ca2+ levels (Li et al., 2016). Other than ADHD, voltage-gated calcium channels have also been implicated in other neuropsychiatric disorders, like schizophrenia, ASD, anxiety, etc. (Nanou and Catterall, 2018; Andrade et al., 2019). Only a few studies investigated the mTOR pathway in ADHD, but both the mTOR and Wnt pathways are implicated in cellular metabolism and energy balance, and emerging data suggests a positive association between ADHD, obesity, and T2DM (Landau and Pinhas-Hamiel, 2019).

Therefore, a growing body of evidence points to a harmful vicious cycle in which impaired Wnt/mTOR-signaling is intertwined with the major AD and ADHD pathophysiology hallmarks at the protein and gene level for each of the pathways and individual diseases, respectively. However, searching for the alterations of those Wnt/mTOR signaling-related parameters found both in AD and ADHD, might reveal a common point of weakness in their respective etiopathogenesis and thus not only help clarify the overlap of sAD and ADHD pathophysiology at the molecular level but also offer possible shared target(s) for disease-modifying drug intervention in their therapy. Elucidation of the Wnt/mTOR signaling dysfunction underlying ADHD-AD overlapping pathophysiology is very important also as a contribution to understanding its role in the pathophysiology of other neurodegenerative disorders like Parkinson’s disease (Huang et al., 2022; Serafino and Cozzolino, 2023).

## 6. Evidence of the link between attention-deficit hyperactivity disorder and Alzheimer’s disease in the context of animal model phenotypic traits

Although most animal models of ADHD and AD do not fulfill all the validation criteria [face, construct, and predictive validity (Willner, 1986)], they are invaluable in elucidating pathomechanism in these disorders. The face validity criteria of both models seem to be satisfied as they relatively faithfully reflect the key symptoms of ADHD (inattention, hyperactivity, and impulsivity) and AD (progressive cognitive deficits). Nevertheless, both models frequently also demonstrate additional phenotypic traits that may or may not reflect the comorbidity/complex presentation in humans. The construct validity of the models is often challenging to determine considering that the etiopathogenesis of ADHD and AD is still not fully understood. In this regard, some pathological and molecular correlates suggest some models may be valid, e.g., most animal models of AD demonstrate accumulation of Aβ and tau hyperphosphorylation. Additionally, a low attentive, low vigilance, and high response disinhibition model of ADHD shows improved vigilance and reduced probability of false alarms upon administration of an agonist of the dopamine D4 receptor (Hayward et al., 2016) associated with adult ADHD (Franke et al., 2012). However, the models also seem to present with features that do not reflect the natural course of AD and ADHD in humans, e.g., Tg2576 animals -fAD model, overexpress a mutant form of APP with a Swedish mutation (KM670/671NL) throughout their life (representing at best ∼1% of familial AD cases) and do not show signs of neuronal loss and neurofibrillary tangles (Irizarry et al., 1997). Similar to that, dopamine transporter (DAT)-knockout mice (ADHD model), show solid face and predictive validity, however, DAT seems to be increased, and not decreased in ADHD patients (Rahi and Kumar, 2021). Finally, ADHD and AD models demonstrate variable positive and negative predictive validity as many drugs that work in animal models (McKean et al., 2021; Dougnon and Matsui, 2022; Kantak, 2022), unfortunately, show little benefits in humans, while some drugs that are successfully used in patients, fail to improve symptoms in some animal models.

Regardless of the aforementioned limitations, animal models sometimes offer unexpected insight into diseases with shared molecular pathobiology offering the opportunity for a better understanding of genetic and environmental risk factors and co-morbidities, as well as the development of new working models and identification of novel (shared) drug targets. Here we propose that a careful evaluation of ADHD and AD models, particularly from the perspective of face validity, provides evidence for potentially shared pathomechanisms and risk factors.

### 6.1. Evidence for Alzheimer’s disease-like behavioral traits in animal models of attention-deficit hyperactivity disorder

As the etiopathogenesis of ADHD remains largely unknown (construct validity), most animal models focus on replicating the symptomatology (face validity), which classically consists of hyperactivity, impulsivity, and inattention (Sontag et al., 2010), i.e., executive dysfunction.

A link between ADHD and AD may be found in the dysfunction of working memory as one of the core executive functions (Roth and Saykin, 2004; Diamond, 2013). In 2000, Baddeley suggested the working memory’s “episodic buffer” as a stage in long-term episodic learning (Baddeley, 2000). A key component of AD symptomatology is the loss of long-term episodic memory (Salkovic-Petrisic et al., 2021), which seems to arise from a deficit in the encoding of new memories (Germano and Kinsella, 2005), implicating a working memory deficit antecedent to long-term memory dysfunction [confirming the “hypothesis 1” by Callahan et al. (2017)]. However, longitudinal studies are necessary. Even though they are sorely lacking in animal models, there is some evidence that the above is precisely the case in at least two different models of ADHD: Sprague–Dawley rats selected for high impulsivity, and the spontaneously hypertensive rat (SHR) model.

In Dellu-Hagedorn et al. (2004) divided 3-month-old Sprague–Dawley rats by impulsivity–at this point, no differences in working memory among groups could be observed (radial arm maze). At middle age (15 months), the difference in impulsivity between groups was retained, but impulsivity decreased overall. In the working memory assessment, the highly impulsive group performed worse in the training phase up until the last day, when it reached the non-impulsive group’s performance. Two years later, Dellu-Hagedorn again divided 3-month-old rats by impulsivity and reproduced these results (Dellu-Hagedorn, 2006). While rats in the hyperactive group made more errors in training, after 6 days they managed to reach the same level of performance as the hypoactive group, indicating a surmountable working memory deficit among the hyperactive animals in this scenario. Another ADHD model, the SHR (Sagvolden and Johansen, 2012), has originally been developed as a model for studying hypertension in 1963 by selectively breeding Wistar-Kyoto rats (Okamoto and Aoki, 1963). The increased locomotor activity of these animals seems to be highly age-dependent, peaking in a juvenile phase around 8 weeks of age (van den Bergh et al., 2006), and again in an older age of 45 weeks (Hendley et al., 1985). Most ADHD research using this model, therefore, focuses on this juvenile period, when no cognitive deficits seem to be present (Langen and Dost, 2011). Young, 7–8-week-old male SHR rats exhibiting increased locomotor activity do not seem to show cognitive impairments in the novel object recognition test (Langen and Dost, 2011). It seems that, in older SHR rats, the hyperactive (in terms of increased locomotor activity) phenotype diminishes as cognitive deficits begin to appear, and spatial memory dysfunction has been observed in 3-month-old SHR rats (Sontag et al., 2013). Another study examining 3- and 7-month-old SHR rats links these deficits to brain IR dysfunction, a phenomenon linked to AD (Grünblatt et al., 2015). A third study working with older (26–30 weeks) SHR rats describes a similar phenotype accompanied by vascular dysfunction in the hippocampus (Johnson et al., 2020), another neuropathology commonly found in AD (Govindpani et al., 2019). While there is also evidence of neuropathology less specific to AD in particular, such as neuroinflammation (Tayebati et al., 2016; Cohen et al., 2019) and increased oxidative stress (Corona, 2020), parenchymal Aβ formation, a hallmark of AD, has also been observed in this model at 20–44 weeks of age with conventional histology and immunohistochemistry, showing an age-dependent increase in parenchymal β-amyloid load (Schreiber et al., 2014).

The aforementioned cognitive deficits developing at a later age provide face validity to the hypothesis that, with age, an ADHD model may serve as a sAD model, and the neuropathological markers found therein thought to be closely related to AD, lend construct validity. Unfortunately, there is little data on therapies in older ADHD model animals to provide a statement on predictive validity.

### 6.2. Evidence for attention-deficit hyperactivity disorder-like behavioral traits in animal models of Alzheimer’s disease

Accumulating evidence suggests that animal models of AD demonstrate some phenotypic traits resembling ADHD. van Swinderen (2007) has shown that mutations in genes involved in short and long-term memory formation (van Swinderen et al., 2009), and memory consolidation (van Swinderen and Brembs, 2010) all result in deficits in attention-like processes in Drosophila melanogaster. Furthermore, the attention deficits were accompanied by well-defined behavioral hyperactivity and phenotypic alterations were successfully alleviated by MPH treatment (van Swinderen and Brembs, 2010) providing evidence for both face and predictive validity (in the context of ADHD-like symptoms). The results from Zhang et al. (2015) illustrate the presence of phenotypic traits of ADHD in AD even more clearly as they observed clear ADHD-like behavior in flies generated as a model of AD. Zhang et al. (2015) generated flies with inducible expression of low levels of human APP and BACE1 to overcome methodological problems associated with the overexpression of AD-associated transgenes. Unexpectedly, inducing low levels of human APP and BACE1 resulted in a phenotype resembling ADHD with: (i) a marked increase in overall motor activity; (ii) male predominance; (iii) carbohydrate-induced aggravation of symptoms; (iv) the phenotype mitigated with age; (v) delayed, but a steep reduction in nocturnal activity; (all strongly indicative of high face validity of the model for ADHD) and; (vi) a reversible reduction in hyperactivity by dextroamphetamine (with the absence of the effect in non-ADHD-like fly controls)—strongly indicative of good predictive validity of the model (Zhang et al., 2015). The construct validity of the proposed model for ADHD remains to be explored, however, based on strong evidence in support of both face and predictive validity, the authors proposed its use for elucidation of the etiopathogenesis of ADHD. The presence of ADHD-like symptoms has also been reported in other, more complex, animal models of AD. For example, Tg2576 mice demonstrate locomotor hyperactivity (Gorman and Yellon, 2010; Bardgett et al., 2011) and increased exploratory behavior (Babic Perhoc et al., 2019) providing some evidence for face validity. Locomotor hyperactivity has also been reported in other transgenic models of AD—e.g., Swedish-APP (Bedrosian et al., 2011), 3xTg-AD (only in male mice) (Pietropaolo et al., 2008; Sterniczuk et al., 2010), Swedish APP on a 129 genetic background (Rustay et al., 2010), TgCRND8 (Walker et al., 2011), APP+PS1 (Arendash et al., 2001), and APP23 (Van Dam et al., 2003) models. Unfortunately, data on ADHD predictive validity in AD models is scarce as most studies utilizing ADHD drugs in AD models focused on cognitive rather than ADHD symptoms. Nevertheless, some reports suggest that treatments that show beneficial effects in ADHD models [e.g., an H3 antagonist ciproxifan (Fox et al., 2002)] also attenuate hyperactive behavior in some models of AD (Bardgett et al., 2011).

Pronounced locomotor hyperactivity of AD animal models has been reported by different groups, as it was recognized as an important confounder precluding valid behavioral analysis. For example, Jankowsky et al. generated a tetracycline-responsive transgenic APP mouse model to study the effects of Aβ production arrest expected from the treatment with inhibitors of secretases. However, mice overexpressing APP throughout their development demonstrated severe locomotor hyperactivity (with 100% penetrance), incompatible with cognitive testing (Jankowsky et al., 2005). Interestingly, the same model has been used to elucidate the effects of the APP overexpression onset on behavioral phenotype, showing that overexpression during early postnatal development resulted in the most pronounced hyperactivity (Rodgers et al., 2012). In contrast, delaying the overexpression of APP until adulthood resulted in a substantial attenuation of the hyperlocomotor phenotype (Rodgers et al., 2012). The latter provides indirect evidence that ADHD and AD may represent two points along a single pathophysiological continuum [i.e., “hypothesis 1” proposed by Callahan et al. (2017)] and suggests that similar noxious stimuli may result in the development of either ADHD or AD depending on the developmental period during which they occur.

Behavioral alterations in the non-transgenic STZ-icv rat model of sAD provide additional evidence in support of the overlapping phenotype of ADHD and AD in animal models with the advantage of the absence of altered gene expression during brain development (as is often the case with transgenic models). In the STZ-icv model, a complex phenotype characterized by a combination of ADHD and AD-like symptoms develops after icv administration of a diabetogenic compound (streptozotocin) in the period in which neural circuits are already fully formed (excluding the possibility of purely neurodevelopmental origin). The STZ-icv model is characterized by a chronic and progressive cognitive decline (Knezovic et al., 2015) (AD face validity) accompanied by neuropathological and metabolic hallmarks of AD [i.e., BIR state (Grünblatt et al., 2007), neuroinflammation (Knezovic et al., 2017), accumulation of Aβ (Salkovic-Petrisic et al., 2011), hyperphosphorylated Tau (Li et al., 2020), mitochondrial dysfunction (Correia et al., 2011), oxidative stress (Sharma and Gupta, 2001), and glucose hypometabolism (Knezovic et al., 2018)] (AD construct validity) (Salkovic-Petrisic et al., 2021). Interestingly, apart from symptoms and molecular alterations resembling AD, the STZ-icv model also develops attentional deficits and locomotor hyperactivity (ADHD face validity). The development of the hyperlocomotor phenotype was first observed by Mayer and Hoyer during the initial behavioral characterization of the STZ-icv model (Mayer et al., 1990), and it was later (similarly as was the case with the transgenic AD models) recognized as an important confounder for behavioral analyses (Homolak et al., 2021). The STZ-icv rats also demonstrate several common features of ADHD: pronounced circadian dysrhythmia [present already in the very early (24–48 h) post-induction period preliminary data—Supplementary Figure 1A; (WASAD Congress, 2021)], increased stress response (Virag et al., 2021), increased social interaction preference [commonly reported in ADHD models—e.g. (Hopkins et al., 2009; Robinson et al., 2012; Gauthier et al., 2015)] (preliminary data—Supplementary Figure 1B), dysfunctional attention dynamics and hesitancy/impulsivity (unpublished preliminary results see Supplementary Figures 1C–F).

In summary, although a more thorough exploration of the pre-cognitive ADHD-like behavioral phenotype of the STZ-icv model is needed, it seems that non-transgenic models of AD may also recapitulate the behavioral aspect of the hypothesized ADHD-AD continuum (Callahan et al., 2017). Experiments testing ADHD predictive validity (mainly the ability of ADHD drugs to counteract ADHD-like symptoms) utilizing the early ADHD-like pre-cognitive stage of the disease in the STZ-icv model may provide critical information to support or reject the hypothesis. Such experiments, planned to be performed in our lab in the near future, will hopefully elucidate this open question. Furthermore, if such experiments confirm the efficacy of ADHD drugs in treating ADHD-like symptoms in the pre-cognitive stage of the disease, they may also provide a platform for testing the hypothesis that a timely introduction of ADHD therapy may delay or even prevent neurodegeneration and cognitive decline in the context of the proposed ADHD-AD pathophysiological continuum.

Altogether, data from both ADHD and AD animal models support the hypothesis of overlapping pathophysiology and the existence of the ADHD-AD continuum. In both cases, the strongest evidence comes from the experiments reporting a complex ADHD/AD phenotype (face validity), however, the data on construct and predictive validity are still scarce. One of the reasons, for the paucity of construct and predictive validity evidence, may be a time-period restricted use of ADHD and AD models and consequent failure to acknowledge the overlapping symptoms and identify them as a rationale to explore molecular patterns of AD in ADHD and vice versa (construct validity), and test ADHD drugs in AD models and vice versa (predictive validity). The animal models of ADHD are mostly examined when the animals are relatively young to faithfully mimic human disease that usually presents at a relatively young age in most patients. Accordingly, most researchers use old animals to model the AD-like phenotype in rodents. Consequently, most research on ADHD models is done at a time point in which cognitive deficits are not yet evident, and most animal studies with AD models fail to acknowledge a potential early ADHD-like stage of the disease. The evidence supporting this hypothesis comes from relatively rare longitudinal studies on ADHD models with behavioral follow-up after dissipation of the hyperactive phenotype (Dellu-Hagedorn, 2006), and the studies on AD that focus on the developmental/early aspect of AD-like pathophysiology that resembles ADHD e.g., overexpression of APP during brain development (Rodgers et al., 2012). A more mindful and less time-restricted approach to behavioral and molecular patterns in models of ADHD and AD may provide additional evidence in support of the overlapping phenotype and motivate researchers to conduct experiments designed to address the lack of construct and predictive validity evidence for the ADHD-AD continuum. Finally, animal research addressing the ADHD-AD continuum hypothesis will inevitably have to deal with the impact of sex on the etiopathogenesis and the progression of the disease actively explored both in the context of ADHD (Ramtekkar et al., 2010; Cortese et al., 2016; Greven et al., 2018) and sAD (Li and Singh, 2014; Toro et al., 2019). In childhood, a great number of females are undiagnosed for ADHD until reaching adulthood which leads to the male prominence that disappears in adult ADHD. Nevertheless, the latter must be acknowledged in a broader context since the exact causes driving the apparent sexual dimorphism of both diseases are yet to be understood and it is possible that at least some of the factors are more cultural and not biological. For example, there is evidence that female children are underdiagnosed in the community setting resulting in biased and overemphasized estimates pertaining to male predominance (Ramtekkar et al., 2010). Furthermore, regardless of the female predominance of sAD, some animal models of the disease demonstrate the male predominance of some ADHD-like phenotypic traits. For example, female 3xTg mice demonstrate less circadian dysrhythmia in comparison with male transgenic animals (Wu et al., 2018).

Summarizing the aforementioned, the analysis of behavioral traits and phenotypes in animal models of ADHD and AD provides additional evidence in favor of the hypothesis of shared etiopathogenesis. Furthermore, considering molecular similarities, primarily related to Wnt and mTOR pathways (described in detail in chapters 5.1 and 5.2), data from animal models support the existence of a common pathophysiological phenomenon involved in the development of both AD and ADHD (Table 2). As studies indicate that manifestation of primary phenotypic characteristics (ADHD predominant—e.g., animal models of ADHD when tested at a young age, some AD models when tested before the development of cognitive deficits; AD-predominant—e.g., animal models of AD in the late phase, some ADHD animal models after locomotor hyperactivity subsides) is dependent on age. Therefore, molecular alterations ensue, longitudinal studies with animal models of both diseases focused on the temporal association of molecular and behavioral alterations will be necessary to fully entangle the association between ADHD and AD.

**TABLE 2:** Wnt/mTOR pathway and behavioral alterations in rodent models of attention-deficit hyperactivity disorder (ADHD) and Alzheimer’s disease (AD).

Animal model	Wnt/mTOR alterations	ADHD-like behavioral alterations	AD-like behavioral alterations
**AD models**			
TgCRND8/APP J20	↑DKK, GSK-3α/β, ↓Wnt, β-catenin (Rosi et al., 2010) ↑DKK, ↓β-catenin (Tong et al., 2022)	locomotor hyperactivity (Walker et al., 2011) circadian dysrhythmia, hyperarousal (Colby-Milley et al., 2015)	+
APP/PS1	↑GSK3β, ↓β-catenin (Xiang et al., 2021) ↓Wnt4 (Yan et al., 2022) ↓Wnt3a, β-catenin, ↑GSK3β (Zhu et al., 2018) ↓mTOR (Ahmad et al., 2017) ↓mTOR (Francois et al., 2014) ↑DKK-1 (Rosi et al., 2010)	locomotor hyperactivity (Arendash et al., 2001)	+
APP23	↓β-catenin, ↑GSK3β (cells isolated from APP23 mice) (He and Shen, 2009)	locomotor hyperactivity (Van Dam et al., 2003)	+
Tg2576	↓mTOR (Ma et al., 2010) ↑DKK-1 (Rosi et al., 2010)	locomotor hyperactivity (Gorman and Yellon, 2010; Rustay et al., 2010; Bardgett et al., 2011; Bedrosian et al., 2011) increased exploratory behavior (Babic Perhoc et al., 2019)	+
3xTg	↓mTOR (Chen et al., 2012) ↓active β-catenin, β-catenin mRNA, ↑inactive β-catenin, GSK3β mRNA, GSK3β activity (Huang et al., 2018)	locomotor hyperactivity (Pietropaolo et al., 2008; Sterniczuk et al., 2010) increased locomotor and exploratory activity (Chen et al., 2013)	+
P301S	↑mTOR (Mencer et al., 2021) ↑non-canonical Wnt (Amal et al., 2019) ↑DKK-1 (Rosi et al., 2010)	increased locomotor activity and exploration (Scattoni et al., 2010; Takeuchi et al., 2011)	+
5xFAD	↓mTOR, ↓β-catenin, ↑GSK3β (Avrahami et al., 2013)	locomotor hyperactivity (Oblak et al., 2021; Sil et al., 2022)	+
STZ-icv mice	↓mTOR (STZ icv 3 months) (Qin G. et al., 2021) ↑mTOR (STZ icv 3 weeks) (El Sayed et al., 2021) ↑β-catenin, ↑GSK3β (STZ 3 mg/kg bilateral hippocampal injection) (Qi et al., 2021) ↓β-catenin, ↓Wnt3a; ↑GSK3β, ↑mTOR (Salem et al., 2021)	decreased locomotor activity and exploration (Qi et al., 2021) increased locomotor activity and exploration (Chen et al., 2013)	+
STZ-icv rats	↑GSK3β (Barilar et al., 2015) ↓mTOR (Chen et al., 2012)	locomotor hyperactivity (Mayer et al., 1990; Homolak et al., 2021), Supplementary Figure 1 (WASAD Congress, 2021) increased stress response (Virag et al., 2021) circadian dysrhythmia [Supplementary Figure 1 (WASAD Congress, 2021)]	+
**ADHD models**			
SHR	↑GSK3β activity, ↓β-catenin (Cheng et al., 2015) ↑GSK3β (Grünblatt et al., 2015)	+	spatial, object, and aversive memory deficits (Sontag et al., 2013; Grünblatt et al., 2015; Johnson et al., 2020)
THRSP OE	↑β-catenin, *Dkk4, Igfbp5, Lrp6;*↓*Wnt7a* (Custodio et al., 2023)	+	recognition memory deficits (Custodio et al., 2018)
Neonatal MSG	↓Wnt3a, β-catenin, ↑GSK3β mRNA (Abu-Elfotuh et al., 2022)	+	spatial memory deficits (Abu-Elfotuh et al., 2022)

Standardly present cognitive alterations in animal models of AD and ADHD-like traits in animal models of ADHD were marked with a “+”. APP, amyloid precursor protein; APP23, B6-Tg/Thy1APP23Sdz; APP/PS1, APP/PS1 double transgenic; DKK, Dickkopf; 5xFAD, APP/PS1, Tg6799, Tg-5xFAD [APP K670_M671delinsNL (Swedish), APP I716V (Florida), APP V717I (London), PSEN1 M146L (A > C), PSEN1 L286V]; GSK3, glycogen synthase kinase 3; mTOR, mammalian target of rapamycin; *Igfbp*, insulin-like growth factor-binding protein; *Lrp*, low-density lipoprotein receptor-related protein; MSG, monosodium glutamate; P301S, Tau PS19Tg (MAPT P301S mutation); PS1, presenilin 1; SHR, spontaneously hypertensive rat; STZ-icv, streptozotocin intracerebroventricular; Tg, transgenic; 3xTg, 3xTg-AD, the Laferla mouse [APP K670_M671delinsNL (Swedish), MAPT P301L, PSEN1 M146V]; Tg2576, hsiao mice, App-Swe, App-sw, APP(sw), APPSwe; TgCRND8, APP(swe/ind) CRND8; THRSP OE, thyroid hormone-responsive gene overexpression.

## 7. Methylphenidate in attention-deficit hyperactivity disorder and Alzheimer’s disease and the link to Wnt/mTOR

Unlike for AD, pharmacological interventions for ADHD have shown a high level of efficacy supported by decades of clinical use and thousands of research studies (Banaschewski et al., 2018; Cortese et al., 2018). In child and adolescent, the efficacy lowering total symptoms following meta-analysis of total 22 studies with 1,603 patients treated with MPH vs. 1,251 placebo controls was estimated to be 0.77 (Faraone and Buitelaar, 2010). For MPH immediate release, the long-term effects of MPH following meta-analysis of seven studies (444 patients) resulted in efficacy of 0.96 and 1.12 for inattention and hyperactivity/impulsivity, respectively (Maia et al., 2017). Moreover, in a recent meta-analysis of 31 studies (804 children), dose-dependent effect of MPH on neurocognitive functioning in children with ADHD showed beneficial effects on all neurocognitive functions (*d* = 0.20–0.73) with linear dosing effects (Vertessen et al., 2022). In adults with ADHD, meta-analysis in eight studies (2,036 patients) found that MPH treatment efficacy of up to 0.58, with increase efficacy of 0.12 for every 10 mg increment of MPH (Castells et al., 2011). MPH is the first-line treatment for ADHD in children and adolescents according to the NICE and the German guidelines (Banaschewski et al., 2018; Cortese et al., 2018). Safety and efficacy have also been demonstrated in adults with ADHD (Castells et al., 2011; Cortese et al., 2018; Solmi et al., 2020), however, despite evidence based guidelines, only half of the European countries have approved the use of MPH for the management of adult ADHD (Chappuy et al., 2020). In AD, MPH has been used as a treatment for apathy (Ruthirakuhan et al., 2018; Kishi et al., 2020; Mintzer et al., 2021), and although it was shown to be a safe and efficacious medication, this use is still off-label. Indeed, in a very small meta-analysis of three double-blind, randomized, placebo-controlled trials (RCTs) investigating MPH treatment of apathy as a primary or secondary outcome in people with AD (*n* = 145), it was shown that MPH treatment is associated with small improvements (in apathy scores) in AD patients (Ruthirakuhan et al., 2018). Interestingly, a slight improvement in cognition measured using mini-mental state examination (MMSE) was observed as well (mean difference of 1.98, CI 1.06–2.91), however, due to a low number of studies, no conclusion could be made. In another meta-analysis including modafinil as a psychostimulant treatment of apathy in AD patients (3 MPH and 1 modafinil study, *n* = 156), an increase in cognitive scores (MMSE) was found alongside improvement of apathy (Kishi et al., 2020). In the recently published RCT with a 6 month follow-up (*n* = 301), MPH improved apathy scores compared to placebo, with improved Alzheimer’s Disease Cooperative Study Clinical Global Impression at 6 months (Mintzer et al., 2021). However, there was no apparent difference in comparison with the placebo group in cognitive performance nor the quality of life (Mintzer et al., 2021). The absence of the effects on cognition in some AD trials may be due to timing, as a small RCT with MCI patients (*n* = 15) demonstrated a beneficial effect of MPH on memory tests (Press et al., 2021). The latter might be due to the neurogenic capacity still available in MCI compared to AD patients.

Despite its well-known clinical efficacy, the exact molecular mechanisms responsible for the beneficial effects of MPH in ADHD remain elusive (Yde Ohki et al., 2020). The effects of MPH are usually attributed to its ability to block the reuptake of dopamine, noradrenaline, and (in low affinity) serotonin to potentiate the effects of monoamines in the synaptic cleft (Quintero et al., 2022). Nevertheless, accumulating evidence suggests that the ability of MPH to stimulate cortical maturation [responsible for its long-term effects (Shaw et al., 2009; Walhovd et al., 2020)] may be mediated by a separate mechanism not related to the inhibition of monoamine transporters (Bartl et al., 2010, 2013b, 2017; Grünblatt et al., 2018; Yde Ohki et al., 2020). It has been reported that MPH can inhibit proliferation and enhance neuronal differentiation both *in vitro* (Bartl et al., 2013b; Grünblatt et al., 2018) and *in vivo* (Lee et al., 2012; Oakes et al., 2019) *via* an unknown molecular mechanism. One possible molecular pathway hypothesized to be responsible for these effects is the Wnt pathway (Yde Ohki et al., 2020). MPH was demonstrated to activate Wnt signaling in three distinct neuronal cell lines (murine stem cells, rat PC12, and human SH-SY5Y cells), independently of its action on the dopamine transporter (considering that the selective dopamine transporter inhibitor GBR-12909 exerted opposite effects) (Grünblatt et al., 2018). The ability of MPH to activate Wnt *in vitro* (Grünblatt et al., 2018) is in line with the reports from *in vivo* studies where the ability of low-dose MPH to promote cell proliferation and survival was associated with increased activity of the Wnt-signaling pathway (Mines and Jope, 2012; Sadasivan et al., 2012; Dela Pena et al., 2013; Oakes et al., 2019; Yde Ohki et al., 2020). The signaling pathway revolving around the mTOR pathway with complementary biological functions to Wnt was proposed as another potential mediator of the effects of MPH on cortical maturation (Yde Ohki et al., 2020). Although mechanisms by which MPH regulates the mTORC1 signaling pathway remain elusive, its ability to modulate the activity of its components (e.g., Akt, GSK3β, p70S6K, 4E-BP1, CREB) has been demonstrated both *in vitro* (Schmitz et al., 2019) and *in vivo* (Warren et al., 2011).

Briefly, MPH is beneficial in treating childhood ADHD while showing beneficial effects in early AD patients, which, following the current evidence, might be also due to its influence on the Wnt/mTOR-pathway. However, longitudinal clinical studies will be required to confirm this hypothesis.

## 8. Conclusion

Following the aforementioned evidence regarding ADHD-AD being a continuum, with several overlapping pathways and mechanisms, it would be of high urgency to move into research over the lifespan in humans and animal models, as well as at the molecular and cellular levels modeling both disorders *in vitro* using e.g., iPSC-derived neural cells (2D and 3D). Moreover, the common Wnt/mTOR pathways that have been altered both in ADHD and AD, and the fact that some drug treatments may influence them, should be studied more in-depth, to explore the possibilities of prevention and/or rescue in these two frequent neurological disorders. Although out of scope of the current hypothesis paper, it should be noted that neurodevelopmental disorders in general also were argued to be linked to sAD (Folsom and Fatemi, 2013; Delhaye and Bardoni, 2021; Ebstein et al., 2021) while neurodegenerative disease with cognitive decline have some overlap to ADHD (Golimstok et al., 2011; Gehricke et al., 2017; Prentice et al., 2021). In particular, keeping in mind all the above and the recent literature proposing normalization of altered Wnt/mTOR signaling as a novel mechanism of action for MPH used as ADHD-treatment, the accumulated evidence provide a convincing argument and an encouragement to explore the idea that a timely introduction of ADHD therapy may delay/prevent neurodegeneration and cognitive decline in AD.

## Data availability statement

The original contributions presented in this study are included in this article/Supplementary material, further inquiries can be directed to the corresponding author.

## Author contributions

EG, JH, PR, CT, and MS-P contributed to the conception and substantiation of the hypothesis. AB, VD, AK, and JO collected the literature evidence on animal modeling. PR, SW, EG, CT, and MS-P collected the literature evidence on clinical aspects. EG wrote the first draft of the manuscript. EG, JH, AB, VD, AK, JO, CT, and MS-P wrote sections of the manuscript. All authors contributed to the manuscript revision, read, and approved the submitted version.
