# Supplementary material for: From attention-deficit hyperactivity disorder to sporadic Alzheimer’s disease—Wnt/mTOR pathways hypothesis
Source: Front Neurosci. 2023 Feb 16;17:1104985. doi: 10.3389/fnins.2023.1104985 (PMC9978448; doi:10.3389/fnins.2023.1104985)
Supplement: Supplementary file 1 [file Image_1.pdf]

Supplementary Material

From Attention-deficit hyperactivity disorder to sporadic Alzheimer's disease - Wnt/mTOR pathways hypothesis

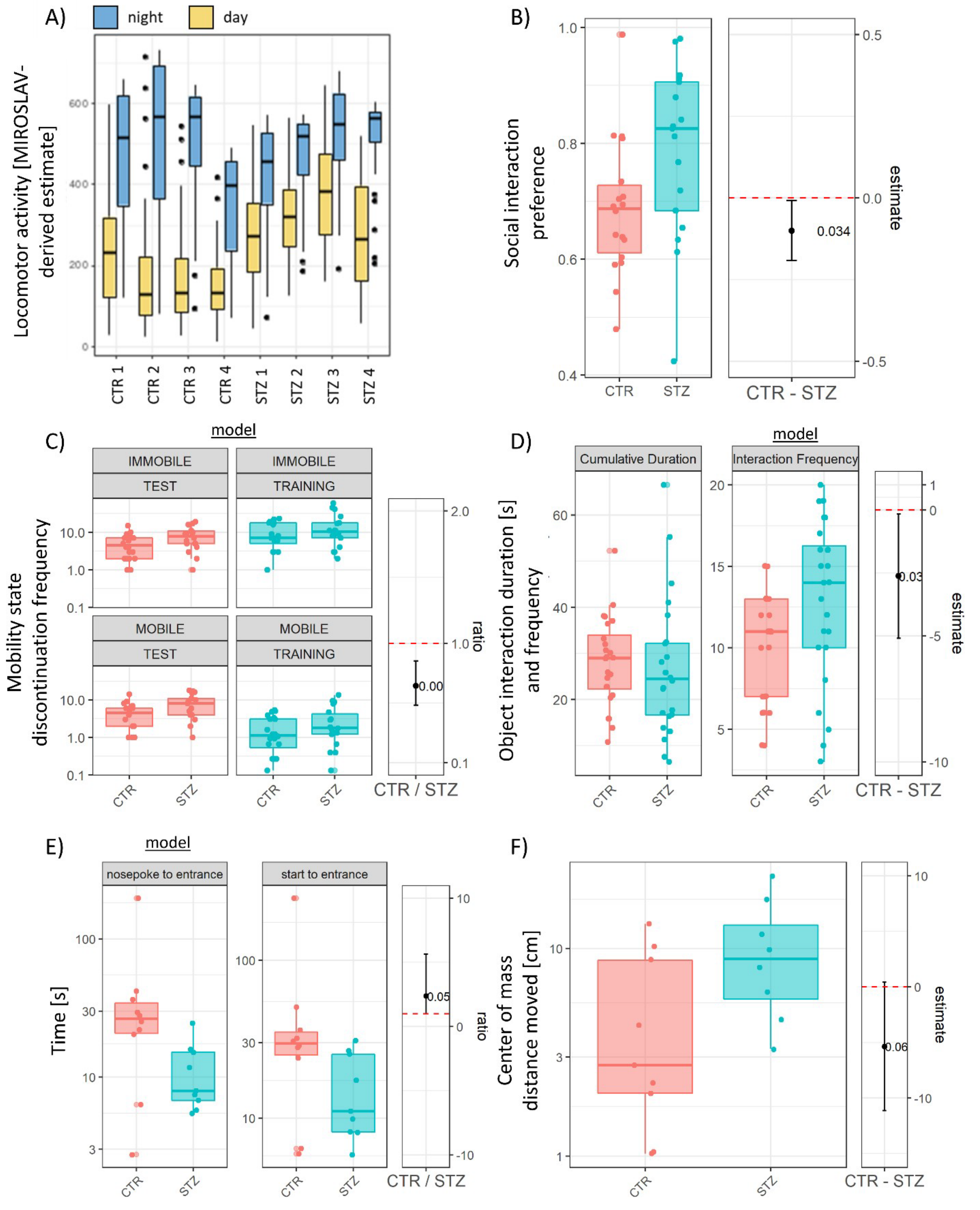

A)
night
day
Locomotor activity [MIROSLAV-derived estimate]
CTR 1
CTR 2
CTR 3
CTR 4
STZ 1
STZ 2
STZ 3
STZ 4
B)
Social interaction preference
CTR
STZ
estimate
0.034
CTR - STZ
C)
model
IMMOBILE
TEST
TRAINING
MOBILE
Mobility state discontinuation frequency
ratio
0.00
CTR / STZ
D)
Cumulative Duration
model
Interaction Frequency
Object interaction duration [s] and frequency
estimate
CTR - STZ
E)
model
nosepoke to entrance
start to entrance
Time [s]
0.05
ratio
CTR / STZ
F)
Center of mass distance moved [cm]
-0.06
estimate
CTR - STZ

Supplementary Figure 1. Preliminary data on the pre-cognitive ADHD-like behavioral phenotype in the intracerebroventricular streptozotocin (STZ-icv)-induced rat model of sporadic Alzheimer's disease (sAD). All data is representative of the STZ-icv model (STZ-icv 3 mg/kg) induced in the 3-month-old male Wistar rats (Department of Pharmacology, University of Zagreb School of Medicine (UZMS), all experiments approved by the UZMS Ethics Committee and the Croatian Ministry of Agriculture (EP 186 /2018; 380-59-10106-18-111/173)). **A)** Pronounced locomotor circadian dysrhythmia in the STZ-icv rat model of sAD assessed with the Multi InfraRed Open-Source Locomotor Activity eValuator (MIROSLAV) system, an open-source platform for non-invasive home cage locomotor activity tracking with passive infrared sensors (Homolak et al., 2021). Data represent pooled values from the first two weeks upon model induction for 8 experimental units (n_{CTR}=4; n_{STZ}=4). Each boxplot represents 672 measurements (24 values/12-hour period). Circadian dysrhythmia is evident already in the very early (24-28h) post-induction period. **B)** Social interaction preference is increased in the STZ-icv rat model of AD 1 month after model induction. Raw data (left) and the effect size (point estimate and 95% confidence intervals of the difference of estimated marginal means derived from the linear model comparing the preference for the social interaction zone in comparison with the control zone in the social interaction test) (right). Social interaction for all animals was assessed using the unfamiliar animal in the modified mouse cage (social interaction object), and the same empty cage in the opposite corner (control object). The arena was divided into quadrants with social interaction zones and the position of the objects was randomized across trials (to account for place preference). All trials were filmed for 5 minutes. Noldus EthoVision XT video tracking software was used for the analysis. N=20/group. **C)** Frequency of mobility states from the novel object recognition task (training and test sessions) in the STZ-icv rat model of AD 1 month after model induction estimated using Noldus EthoVision XT video tracking software (left). The effect size (point estimate and 95% confidence intervals of the ratio of estimated marginal means derived from the linear model with the mobility state and session type (training vs test) introduced as covariates (right). The STZ-icv rats demonstrate greater discontinuation of both the transition from the immobile to the mobile state (movement initiation) and the transition from the mobile to the immobile state (movement cessation) in the same period (5 minutes). In summary, the STZ-icv rats demonstrate reduced action consistency and persistence resembling ADHD. N=20/group **D)** Object interaction duration and frequency derived from the novel object recognition task in the STZ-icv rat model of AD 1 month after model induction. Raw data (left) and the effect size (point estimate and 95% confidence intervals of the difference of estimated marginal means derived from the linear model with object interaction frequency used as the dependent variable. The STZ-icv rats demonstrate slightly reduced total object interaction time, but increased object interaction frequency (increased number of attempts to investigate object) in the training sessions of the novel object recognition task. More attempts to investigate objects (for a shorter period) are in line with reduced action consistency and persistence resembling ADHD. N=20/group. **E)** Behavior of the STZ-icv rats during the habituation in the passive avoidance task demonstrating increased impulsivity in the STZ-icv rats (decreased "decision-making" time). The STZ-icv rats enter the unknown arena faster than the controls. Raw data (left) and the effect size (point estimate and 95% confidence intervals of the ratio of estimated marginal means derived from the linear model with nose poke-to-entrance time used as the dependent variable) (right). N=10/group. **F)** Distance moved in the light chamber of the passive avoidance task presented in E. The STZ-icv rat model of AD demonstrates greater exploration (i.e. distance moved) before entering the unknown arena (without adjusting for the reduced entrance time). Raw data (left) and the effect size (point estimate and 95% confidence intervals of the difference of estimated marginal means derived from the linear model with the center of mass distance moved used as the dependent variable) (right). N=9 controls (CTR); N=8 STZ. In all figures, boxplots were used to demonstrate the distribution of a continuous variable. Upper and lower hinges correspond to the first and third quartiles and whiskers

extend from the hinges to the largest/smallest value no further than 150% of the inter-quartile range (data beyond whiskers were taken into calculation but represented as the outlying data points).

Homolak, J., Virag, D., Babić Perhoč, A., Kodvanj, I., Joja, M., Knezovic, A., et al. (2021). Too nervous to sleep? – novel behavioral aspects of the intracerebroventricular streptozotocin-induced rat model of sporadic Alzheimer's disease. in (Vienna: Springer). doi: 10.1007/s00702-021-02422-z.
